# A Population Pharmacokinetic Analysis Shows that Arylacetamide Deacetylase (AADAC) Gene Polymorphism and HIV Infection Affect the Exposure of Rifapentine

**DOI:** 10.1128/AAC.01964-18

**Published:** 2019-03-27

**Authors:** Jose Francis, Simbarashe P. Zvada, Paolo Denti, Mark Hatherill, Salome Charalambous, Stanley Mungofa, Rodney Dawson, Susan Dorman, Nikhil Gupte, Lubbe Wiesner, Amina Jindani, Thomas S. Harrison, Adeniyi Olagunju, Deirdre Egan, Andrew Owen, Helen M. McIlleron

**Affiliations:** a Division of Clinical Pharmacology, Department of Medicine, University of Cape Town, Cape Town, South Africa; b South African Tuberculosis Vaccine Initiative (SATVI), Institute of Infectious Disease and Molecular Medicine and Division of Immunology, Department of Pathology, University of Cape Town, Cape Town, South Africa; c Aurum Institute for Health Research, Johannesburg, South Africa; d Harare City Health Department, Ministry of Health, Harare, Zimbabwe; e Division of Pulmonology, Department of Medicine, University of Cape Town Lung Institute, Cape Town, South Africa; f Johns Hopkins University School of Medicine, Baltimore, Maryland, USA; g Institute for Infection and Immunity, St. George’s, University of London, London, United Kingdom; h Faculty of Pharmacy, Obafemi Awolowo University, Ile-Ife, Nigeria; i Department of Pharmacology and Therapeutics, University of Liverpool, Liverpool, United Kingdom

**Keywords:** *AADAC*, *SLCO1B1*, pharmacogenetics, population pharmacokinetics, rifapentine, tuberculosis

## Abstract

Rifapentine is a rifamycin used to treat tuberculosis. As is the case for rifampin, plasma exposures of rifapentine are associated with the treatment response. While concomitant food intake and HIV infection explain part of the pharmacokinetic variability associated with rifapentine, few studies have evaluated the contribution of genetic polymorphisms. We evaluated the effects of functionally significant polymorphisms of the genes encoding OATP1B1, the pregnane X receptor (PXR), constitutive androstane (CAR), and arylacetamide deacetylase (AADAC) on rifapentine exposure. Two studies evaluating novel regimens among southern African patients with drug-susceptible pulmonary tuberculosis were included in this analysis. In the RIFAQUIN study, rifapentine was administered in the continuation phase of antituberculosis treatment in 1,200-mg-once-weekly or 900-mg-twice-weekly doses. In the Daily RPE study, 450 or 600 mg was given daily during the intensive phase of treatment. Nonlinear mixed-effects modeling was used to describe the pharmacokinetics of rifapentine and to identify significant covariates. A total of 1,144 drug concentration measurements from 326 patients were included in the analysis. Pharmacogenetic information was available for 162 patients. A one-compartment model with first-order elimination and transit compartment absorption described the data well. In a typical patient (body weight, 56 kg; fat-free mass, 45 kg), the values of clearance and volume of distribution were 1.33 liters/h and 25 liters, respectively. Patients carrying the AA variant (65.4%) of *AADAC* rs1803155 were found to have a 10.4% lower clearance. HIV-infected patients had a 21.9% lower bioavailability. Once-weekly doses of 1,200 mg were associated with a reduced clearance (13.2%) compared to that achieved with more frequently administered doses. Bioavailability was 23.3% lower among patients participating in the Daily RPE study than in those participating in the RIFAQUIN study. This is the first study to report the effect of *AADAC* rs1803155AA on rifapentine clearance. The observed increase in exposure is modest and unlikely to be of clinical relevance. The difference in bioavailability between the two studies is probably related to the differences in food intake concomitant with the dose. HIV-coinfected patients had lower rifapentine exposures.

## Abstract (precis)

Rifapentine is a rifamycin used to treat tuberculosis. As is the case for rifampin, plasma exposures of rifapentine are associated with the treatment response.

## INTRODUCTION

Rifamycins play a key role in the multidrug treatment of tuberculosis. Their sterilizing activity is exposure dependent (1–3). Rifapentine was approved by the Food and Drug Administration (FDA) in 1998 for the treatment of pulmonary tuberculosis (3, 4). Rifapentine pharmacokinetics are influenced by age, weight, dosing pattern, human immunodeficiency virus (HIV) infection, and sex (5, 6). Rifapentine is less rapidly absorbed than rifampin, with peak plasma concentrations being reached within 5 h. Concomitant food intake markedly increases its absorption; the extent of rifapentine absorption increased by 33 to 86% when given with meals (7). Rifapentine has a half-life of approximately 12 h in humans (8, 9). With its long half-life and excellent sterilizing activity, rifapentine is an attractive alternative to rifampin and is increasingly used to treat active tuberculosis and latent infection. However, there is marked interpatient variability in rifamycin pharmacokinetics (10). The primary metabolic pathways for rifapentine involve deacetylation to the primary enzymatic metabolite, 25-desacetyl rifapentine, which is mediated by human arylacetamide deacetylase (AADAC), and nonenzymatic hydrolysis, resulting in the formation of the secondary metabolites 3-formyl rifapentine and 3-formyldesacetyl rifapentine (11). The protein binding of rifapentine is estimated to be about 98% (3, 12). Like other rifamycins, rifapentine induces its own metabolism (9).

Previously published data indicate that single nucleotide polymorphisms (SNPs) in the solute carrier organic anion transporter 1B1 (SLCO1B1) gene encoding the OATP1B1 transmembrane receptor affect rifampin concentrations (13, 14). The *SLCO1B1* rs4149032 C > T polymorphism, found in 70% of South Africans with tuberculosis living in Cape Town, was associated with 20% and 28% reductions in rifampin bioavailability in heterozygotes and homozygotes, respectively (14). Rifamycins are also substrates of the drug efflux pump P glycoprotein, coded for by the polymorphic *ABCB1* gene (15), and are metabolized mainly by polymorphic human arylacetamide deacetylase (AADAC) (16). Human rifamycin exposures are also modulated by the pregnane X receptor (PXR) and constitutive androstane (CAR) nuclear receptors (17). Since the development of resistance to rifamycins and their bactericidal effects are related to rifamycin concentrations, SNPs substantially influencing rifamycin concentrations may be of therapeutic importance. Little is known about the pharmacogenetic correlates of rifapentine pharmacokinetics, which may potentially help in finding the optimal dose of rifapentine. Therefore, the aim of this study was to determine the effect of polymorphisms of *SLCO1B1*, *PXR*, *CAR*, and *AADAC* on rifapentine pharmacokinetics.

## RESULTS

A total of 326 patients were included in the study and contributed a total of 1,151 concentrations-time points. Only 7 concentrations were below the lower limit of quantification (LLOQ) and were omitted from the analysis. The median body weight and the median age of the study participants were 56 kg and 32 years, respectively. All demographic characteristics are summarized in Table 1.

**TABLE 1:** Demographic and clinical characteristics of patients

Demographic or clinical characteristic	Values for patients from the following study:				
	Daily RPE, 450 mg (*n* = 44)	Daily RPE, 600 mg (*n* = 41)	RIFAQUIN, 900 mg (*n* = 116)	RIFAQUIN, 1,200 mg (*n* = 125)	Overall (*n* = 326)
No. of samples for PK^*a*^ analysis	166	130	416	432	1,144
No. of males/no. of females	33/11	32/9	72/44	81/44	218/108
No. (%) of HIV-positive patients	6 (13.6)	7 (17.1)	30 (25.9)	16(12.8)	59(18.1)
Median (range) age (yr)	29 (19–61)	29 (18–63)	31 (19–64)	34 (19– 80)	32 (18–80)
Median (range) wt (kg)	55 (45–79)	55 (45–94)	55 (38–77)	57 (38– 78)	56 (38–94)
Median (range) FFM (kg)	47 (32–58)	47 (32–56)	45 (27–62)	45 (27–60)	45 (27–62)

a PK, pharmacokinetic.

**FIG 1:**
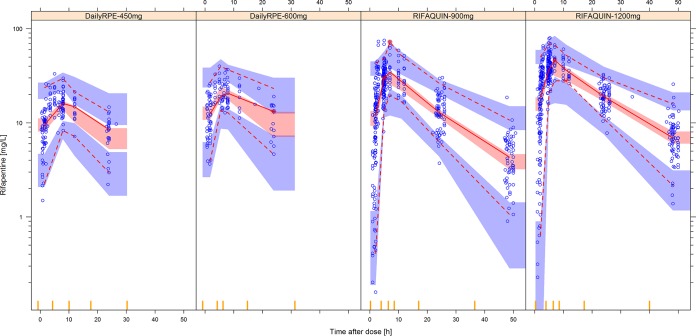
Visual predictive check (VPC) for the final rifapentine population pharmacokinetic model in log scale, stratified according to the different dose groups in the analysis. The lower, middle, and upper solid lines are the 2.5th, 50th, and 97.5th percentiles of the observed plasma concentration, respectively. The shaded areas are the 95% confidence intervals for the same percentiles, obtained from resimulations of the same trial.

The population pharmacokinetics of rifapentine were well described by a one-compartment model with first-order elimination and transit compartment absorption. Fat-free mass (FFM) was found to be the best size descriptor for clearance (change in the NONMEM objective function value [ΔOFV], 93 points [*P* < 0.001] when including FFM for allometric scaling on clearance and 23 points better than when using body weight), and total body weight was found to be the best size descriptor for the volume of distribution (ΔOFV, 20; *P* < 0.001). The absorption of rifapentine was described using a series of transit compartments, which significantly improved the model with respect to the use of simple first-order absorption (ΔOFV, 421; *P* < 0.001). In a typical patient (FFM, 46 kg; weight, 56 kg), the values of clearance and volume of distribution were 1.33 liters/h and 25 liters, respectively. Final parameter estimates (shown in Table 2) were in agreement with the previously published results (6, 18), and a visual predictive check (VPC) of the final model is shown in Fig. 1.

**TABLE 2:** Final parameter estimates for rifapentine population pharmacokinetic model^*a*^

Parameter	Estimate		Variability	
	Value	95% CI^*c*^	% CV	95% CI
CL^*b*^ (liters/h)	1.33	1.14, 1.54	23.0 (IIV)	17.7, 28.6
*V*^*b*^ (liters)	25	21.9, 28.4	12.8 (IIV)	8.8, 17.4
*k_a_* (h^−1^)	0.814	0.568, 1.26	48.9 (IOV)	36.4, 59.8
MTT (h)	1.47	1.20, 1.78	37.4 (IOV)	28.3, 48.6
NN	10.2	6.70, 14.0		
*F*	1 (fixed)		20.3 (IOV)	14.9, 26.4
Proportional residual error (%)	9.56	7.09, 13.2		
Additive residual error (mg/liter)	0.247	0.143, 0.401		
Effect of HIV^+^ on *F* (%)	−21.9	−33.2, −6.64		
Effect of group on 1,200-mg dose in RIFAQUIN study on CL (%)	−13.2	−22.8, −4.36		
Effect of Daily RPE study on *F* (%)	−23.3	−35.6, −9.25		
*AADAC* rs1803155 (AA) effect on CL (%)	−10.4	−17.3, −3.53		

a CL, oral clearance; *V*, apparent volume of distribution in the central compartment; *k_a_*, first-order absorption rate constant; MTT, absorption mean transit time; NN, number of hypothetical transit compartments; *F*, oral bioavailability; HIV^+^, human immunodeficiency virus positivity; *AADAC*, arylacetamide deacetylase gene; IIV, interindividual variability; IOV, interoccasion variability; CV, coefficient of variation; CI, confidence interval.

b The typical values of clearance and volume of distribution reported for a patient with a body weight of 56 kg and FFM of 46 kg.

c The 95% confidence interval of parameter estimates was obtained with sampling importance resampling (SIR; *n* = 1,000) of the final model.

Of 326 patients, pharmacogenetic data were available for 162 (49.7%), all of whom were enrolled from South African sites. The distribution of genotype and allele frequencies are presented in Table 3. *SLCO1B1* rs2306283 and *AADAC* rs1803155 variant alleles were found in 82% of patients, whereas the *NR1I2* rs2472677 and *NR1I2* rs1523130 variant alleles existed at a low overall frequency of 33.5% and 16.4%, respectively. In keeping with our previous findings among South Africans in Cape Town (14), the *SLCO1B1* rs4149032 variant allele frequency was found to be 0.75 (Table 3).

**TABLE 3:** Observed genotype and allele frequency of single nucleotide polymorphisms in the study^*a*^

Genotype	Genotype (frequency^*b*^)			Allele (frequency)	
*SLCO1B1* A > G rs2306283	AA (8, 4.94)	AG (43, 26.5)	GG (111, 68.5)	A (0.18)	G (0.82)
*SLCO1B1* C > T rs4149032	CC (15, 9.26)	CT (52, 32.1)	TT (95, 58.6)	C (0.25)	T (0.75)
*NR1I2* C > T rs2472677^*c*^	CC (71, 44.1)	CT (72, 44.7)	TT (18, 11.2)	C (0.67)	T (0.34)
*NR1I2* T > C rs1523130	TT (116, 71.6)	CT (39, 24.1)	CC (7, 4.3)	T (0.84)	C (0.16)
*AADAC* G > A rs1803155	GG (3, 1.85)	GA (53, 32.7)	AA (106, 65.4)	G (0.18)	A (0.82)

a Data are for 162 patients, unless indicated otherwise.

b The data in parentheses represent the number, percent, of patients.

c Data are available only for 161 patients.

After screening and inclusion of genetic information (and imputation of the missing genotype with a mixture model), patients homozygous for the *AADAC* rs1803155 AA polymorphism were found to have a 10.4% lower clearance of rifapentine than subjects that were rs1803155 GG or GA (ΔOFV, 6.2; *P* = 0.013). Initially, the three categories of rs1803155 (AA, GA, GG) were analyzed as separate groups to estimate the respective effects of GA and GG. However, the estimated effects were similar for GG and GA, and when combined, the model goodness of fit (GOF) was not affected. Using the principle of parsimony, we decided to use the simpler model, as the effects of GG and GA were not statistically significant. The other pharmacogenetic variants did not affect the pharmacokinetic parameters.

Patients infected with HIV were found to have a 21.9% lower bioavailability (ΔOFV, 42; *P* < 0.001). The patients who were treated with high 1,200-mg doses of rifapentine tended to have clearance reduced by 13.2% compared to the clearance for the patients in the other dose groups (ΔOFV, 17; *P* < 0.001). The pharmacokinetic differences between the two studies were explored, and it was found that the bioavailability of rifapentine in the Daily RPE study was 23.3% lower than that in the RIFAQUIN study (ΔOFV, 59; *P* < 0.001). The pharmacogenetic covariates other than the *AADAC* rs1803155 polymorphism did not have significant effects on the pharmacokinetic parameters.

## DISCUSSION

The present study is the first to investigate the influence of various plausible physiologically relevant candidate gene polymorphisms on rifapentine pharmacokinetics. We developed a population pharmacokinetic model of rifapentine which was consistent with that developed in previous studies and tested the effect of genotype information on the pharmacokinetic parameters. We showed that the *AADAC* rs1803155 polymorphism is associated with rifapentine clearance. Subjects carrying the AA genotype had a 10.4% lower clearance than those carrying AG or GG, thus leading to increased rifapentine exposure. The low clearance due to this polymorphism is consistent with the findings of previous studies reporting the decreased activity of *AADAC* due to the presence of the variant allele (19). The majority of patients in our study had the *AADAC* rs1803155 AA variant allele, which occurred at a frequency of 0.82, and 65% were homozygous for the single nucleotide polymorphism, which could, in part, account for the relatively high rifapentine exposures described. The polymorphism occurs at lower frequencies of 0.50 to 0.64 in European American, African American, Korean, and Japanese populations (19). Another study identified lower rifapentine concentrations in black Africans, but the influence of pharmacogenetic factors, which might account for the difference in the genotype frequencies between the populations, was not explored (20), whereas Sloan et al., who explored the influence of *AADAC* gene polymorphisms on rifampin pharmacokinetics in Malawian patients, did not identify a significant relationship (21).The prevalence of variant genotypes is different between African ethnic groups and may be the reason for this contrasting effect. As only 3 of 162 patients had rs1803155 GG, no meaningful separate estimate of clearance for this genotype could be obtained. In further attempts to explain the variability in rifapentine pharmacokinetics, we explored the effects of several polymorphisms of drug transporters and transcriptional regulators. The choice of polymorphisms was based on those previously described to affect drug disposition and also previous pharmacogenetic studies conducted with rifampin. Interestingly, we could not detect the effect of the *SLCO1B1* rs4149032 polymorphism on the pharmacokinetics of rifapentine, even with a carrier-no carrier approach. The frequency of *SLCO1B1* in our cohort was 0.75, which is in agreement with previous findings in South African patients from the Cape Town region. Similarly, we did not find a statistically significant effect associated with *SLCO1B1* rs2306283, which existed in our study population at a frequency of 0.82. *SLCO1B1* polymorphisms have been reported to be associated with low rifampin levels (13, 14), and the lack of an effect on rifapentine may suggest differences in the absorption, distribution, metabolism, and excretion of the two drugs. It may be that this transporter does not play a major role in the pharmacokinetics of rifapentine or that the variant allele is associated with greater induction by rifampin. We did not observe an effect due to polymorphisms of the transcriptional regulators. This could be due to the activation of PXR or CAR by rifapentine, which may have overridden any constitutive effects.

Additionally, we found that HIV-infected patients have a lower bioavailability of rifapentine. While the association of HIV infection with antituberculosis drug exposures is inconsistent, our findings for rifapentine are consistent with those from recent studies (22–24). The data available were not sufficient to identify potential interactions of rifapentine with the various antiretroviral drugs prescribed concomitantly.

Patients in the higher-dose group (1,200 mg given once weekly) had increased exposure in the current study, contrary to the findings of Savic et al., who described a decrease in the bioavailability of rifapentine with increased dose (6). The reduced dosing frequency in this group may have led to reduced autoinduction and, thus, increased exposure.

Previous reports demonstrated that exposure to rifamycins is reduced in males due to a higher FFM/body weight ratio (25). The study by Langdon et al. described a 35% reduction in the clearance of 25-desacetyl rifapentine among females (5). In the present analysis, as allometric scaling with FFM accounted for the variability associated with sex, we did not observe any outstanding effects of sex. There was a difference in bioavailability between the two studies included in this analysis. This may be due to differences in food intake with the dose. Rifapentine absorption is strongly enhanced when it is administered with food (7). The finding that the Daily RPE study had a lower bioavailability may arise from the fact that meals with the dose were not standardized, in contrast to the RIFAQUIN study, where a standard meal was provided throughout the study.

To conclude, our study is the first to show that the *AADAC* rs1803155 (AA) genotype is associated with lower rifapentine clearance, leading to increased rifapentine exposure. This effect should be confirmed in a larger independent analysis. The pharmacogenetic association was modest compared to the study effect, which is likely linked to differences in the pattern of food use across the studies and highlights the importance of food intake recommendations both when the drug is used in a programmatic setting and when its pharmacokinetics are investigated. Additionally, we found that rifapentine exposure was lower in HIV-infected patients, a finding that is consistent with the findings of previous studies and that warrants further investigation to assess whether dose adjustment strategies should be considered. Lastly, patients dosed with 1,200-mg-once-weekly doses had lower clearance, possibly as a result of less pronounced autoinduction.

## MATERIALS AND METHODS

### Study population.

This analysis was performed on patients diagnosed with pulmonary tuberculosis from two clinical studies: the phase III RIFAQUIN study (registration number ISRCTN44153044) (26) and the two-stage activity-safety study of daily rifapentine (27), referred to here as the Daily RPE study (ClinicalTrials.gov registration number NCT00814671). A subset of participants from these studies provided their consent for us to assess the effect of genetic polymorphisms of nuclear receptors, drug-metabolizing enzymes, and drug transporters on the pharmacokinetics of rifapentine.

The RIFAQUIN study included two experimental arms in which patients were dosed with daily moxifloxacin, rifampin, pyrazinamide, and ethambutol for 2 months, followed by a continuation phase with either 4 months of 1,200 mg rifapentine once weekly together with 400 mg moxifloxacin or 2 months of 400 mg moxifloxacin twice weekly with 900 mg rifapentine. The RIFAQUIN study was conducted at sites in the Western Cape and Gauteng regions of South Africa and in Harare, Zimbabwe. The doses of rifapentine and moxifloxacin were taken with 240 ml of water 15 min after a light meal of 2 hard-boiled eggs with bread. During the 4th month of treatment, blood samples were drawn for determination of plasma rifapentine concentrations. The pharmacokinetic assessment involved rich sampling (with samples drawn at pre-dose and 1, 2, 3, 5, 7, 10, 12, 26, and 50 h after dosing) or sparse sampling (with samples drawn at about 2, 5, and 24 or 48 h after dosing).

The Daily RPE study was open label and had two experimental arms. Patients with pulmonary tuberculosis were randomized to 450 or 600 mg rifapentine daily, which replaced 600 mg rifampin during the intensive phase of standard therapy. The study participants were recruited in the Western Cape, South Africa. The patients were advised to take the required rifapentine dose with food, but no standardized meal was provided during the study, and no accurate details about food intake with the dose were recorded. Pharmacokinetic sampling was performed at approximately 1 month after starting therapy, and samples were obtained with either intensive sampling (with samples drawn at pre-dose and at 0.75, 1.5, 3.5, 5, 12, and 24 h after dosing) or sparse sampling (with samples drawn 0.5 to 2 h and 5 to 8 h after dosing). Separate written informed consent for the pharmacogenetic study was obtained from participants retrospectively. The pharmacogenetic study was reviewed and approved by the Research Ethics Committee of the University of Cape Town and the University of the Witwatersrand.

### Drug determination.

Plasma rifapentine concentrations were determined with a validated liquid chromatography-tandem mass spectrometry (LC-MS/MS) assay developed in the Division of Clinical Pharmacology, University of Cape Town. Samples were processed with a protein precipitation extraction method using rifaximin as the internal standard, followed by high-performance liquid chromatography with MS/MS detection using an AB Sciex API 3200 instrument. The analyte and internal standard were monitored at mass transitions of the protonated precursor ions *m/z* 877.3 and *m/z* 786.3 to the product ions *m/z* 845.4 and *m/z* 754.1 for rifapentine and rifaximin, respectively. The calibration curves fit quadratic (weighted by 1/concentration) regressions over the range of 0.156 to 40.0 mg/liter for rifapentine. The accuracies for the rifapentine assay were 103.9%, 102.8%, and 97.5% at the low, medium, and high quality control levels, respectively, during interbatch validation. The lower limit of quantification (LLOQ) was 0.156 mg/liter.

### SNP genotyping.

Genomic DNA was extracted from 200 μl whole blood using a QIAamp DNA minikit (Qiagen, Inc., Valencia, CA) in accordance with the manufacturer’s protocol. DNA was quantified spectrophotometrically using a NanoDrop spectrophotometer (Thermo Fisher Scientific Inc., Wilmington, DE) before storage at −20°C. Genotyping was performed by real-time PCR on a DNA Engine Chromo4 system (Bio-Rad Laboratories, Inc., Hercules, CA). The PCR protocol involved an initial denaturation step at 95°C for 15 min, followed by 50 cycles of amplification at 95°C for 15 s and a final annealing at 60°C for 1 min. TaqMan genotyping master mix and assays for *SLCO1B1* rs2306283 (SNP identifier C_1901697_20), *SLCO1B1* rs4149032 (C_1901709_10), *NR1I2* rs2472677 (C_26079845_10), *NR1I2* rs1523130 (C_9152783_20), and *AADAC* rs1803155 (C_8911003_1_) were obtained from Thermo Fisher Scientific (Waltham, MA). Allelic discrimination plots and genotype assignments were performed using Opticon Monitor (version 3.1) software from Bio-Rad Laboratories.

### Pharmacokinetic analysis.

Rifapentine plasma concentration-time data were analyzed using a nonlinear mixed-effects model implemented in NONMEM (version 7.4.2) software (28). The execution of runs was through the Perl-speaks-NONMEM, Pirana, and graphical diagnostics were created using Xpose (version 4.6.0) and R software (29, 30). Estimation of typical population pharmacokinetic parameters, along with their random interindividual variability (IIV) and interoccasion variability (IOV), was performed using a first-order conditional estimation method with the ε-η interaction (FOCE INTER). A lognormal distribution was assumed for IIV and IOV, and a combined additive and proportional model for the residual unexplained variability (RUV) was evaluated. Various structural models including a one- or two-compartment distribution with first-order elimination and first-order absorption with or without a lag time or transit compartment absorption were tested (31). The influence of genetic polymorphisms on the rifapentine pharmacokinetics for patients with an unknown genotype was identified using mixture modeling (32). The effect of the genotype was first tested using the method EXTRA, which not only estimates the association only for patients with available genetic information but also estimates an additional covariate effect for patients with the unknown genotype. Subsequently, the MIX method to impute values using mixture modeling was applied to include the patient with unknown genotype to strengthen the robustness of the findings (32). Model selection was based on changes in the NONMEM objective function value (ΔOFV) and visual inspection of conditional weighted residuals (CWRES) versus time, visual predictive checks (33), and basic goodness-of-fit (GOF) plots. During model development, physiological plausibility and the precision of the parameter estimates were also considered. The model parameters of the final model were evaluated for their precision using the sampling importance resampling (SIR) method (34).

Allometric scaling was applied to clearance (CL) and the volume of distribution (*V*) to adjust for the effect of body size, as described by Anderson and Holford (35). Fat-free mass (FFM) and fat mass (FAT) were tested as alternative size predictors instead of total body weight through allometric scaling (35, 36). After the inclusion of allometric scaling, potential demographic, study site-specific, and pharmacogenetic covariates were screened by inspecting parameter-versus-covariate plots and then tested in the model using drops in the objective function value (which was assumed to be χ^2^ distributed and, thus, in which a 3.84-point drop was considered significant at a *P* value of <0.05 for the inclusion of a single parameter) while scrutinizing the physiological plausibility of the effect (37).
